# Effects of Wise Intervention on Perceived Discrimination Among College Students Returning Home From Wuhan During the COVID-19 Outbreak

**DOI:** 10.3389/fpsyg.2021.689251

**Published:** 2021-06-07

**Authors:** Ting Lu, Zihan Guo, Hao Li, Xinyu Zhang, Zhihong Ren, Weiping Yang, Liuqing Wei, Ling Huang

**Affiliations:** ^1^Department of Psychology, Faculty of Education, Hubei University, Wuhan, China; ^2^Key Laboratory of Adolescent Cyberpsychology and Behavior, Key Laboratory of Human Development and Mental Health of Hubei Province, Ministry of Education, School of Psychology at Central China Normal University, Wuhan, China

**Keywords:** COVID-19, perceived discrimination, perceived social support, wise intervention, mental health

## Abstract

At the beginning of the coronavirus disease 2019 (COVID-19) outbreak, college students returning home from Wuhan, Hubei Province, experienced various degrees of discrimination. This study first investigates perceived discrimination among college students returning home from Wuhan. Then, an experimental method is used to investigate the effectiveness of an intervention designed to reduce the perceived discrimination among those who returned to towns outside of Hubei Province. A total of 63 college students participated in the experiment. In the experimental group (*N* = 31), a wise intervention based on reading and writing was adopted to intervene in perceived discrimination among the participants. The results showed that the perceived discrimination among students returning from Wuhan to towns outside of Hubei Province was significantly higher than that among students returning to towns within Hubei Province. The wise intervention reduced the perceived discrimination in the experimental group but not in the control group. Further analysis found that perceived social support fully mediated the relationship between the intervention and perceived discrimination. These results provide insights on how the content of intervention (perceived social support) and the form of intervention (wise intervention) can prevent the occurrence of psychological problems in epidemic situations.

## Introduction

The coronavirus disease 2019 (COVID-19) was first reported in Wuhan in December 2019. After that, the disease spread throughout Hubei Province and other parts of China, and then to numerous other countries (Shaukat et al., 2020). This pandemic has had an immense adverse impact on the physical and mental health of the population in China (Ju et al., 2020). Although great efforts have been undertaken to curb the epidemic, the significant morbidity and mortality of this virus triggered an unprecedented level of panic and fear in the communities (Qiu et al., 2020; Salari et al., 2020), leading to adverse mental health outcomes such as anxiety, depression, and post-traumatic stress symptoms (Ahmed et al., 2007). In addition, infectious disease outbreaks have also been associated with stigma (Otu et al., 2020), and anxiety and fear related to an infection can lead to acts of discrimination (Usher et al., 2020), which could further exacerbate existing health problems or trigger new ones (Lee et al., 2016; Lian and Kawachi, 2020).

Discrimination can be defined as the prejudicial and/or distinguishing treatment of an individual based on their actual or perceived membership or certain characteristics (Skosireva et al., 2014). Perceived discrimination is a kind of subjective experience relative to objective discrimination and involves the perception of the individual of being treated differently or unfairly due to belonging to a group (such as race or illness) (Major et al., 2002). Stress resulting from perceived discrimination can have a negative impact on physical and mental health and may increase the likelihood of health-threatening behaviors (Williams et al., 1997). For example, studies have shown that unfair treatment and perceived discrimination are risk factors for poor health (Pascoe and Smart Richman, 2009), and perceived discrimination can also lead to psychological disorders such as depression and eating disorders (Kim et al., 2019; Kelly et al., 2020).

In the context of COVID-19, psychological intervention for individuals who might be discriminated against (such as those in high-risk areas) is of great importance for both the prevention and treatment of psychological problems. Researchers have made many interventions against discrimination (Evans-Lacko et al., 2012; Li et al., 2019). However, most of these interventions targeted the removal of the drivers of stigma or the shifting of norms and policies that facilitate the stigmatization process (Stangl et al., 2013) by aiming to reduce stigma and discrimination against people with health conditions (Stangl et al., 2019), i.e., these interventions aimed to reduce discrimination against specific groups. However, in general, interventions that target the perceived discrimination of the discriminated groups are still lacking.

Among a variety of factors influencing perceived discrimination, perceived social support may be key. Perceived social support is the support subjectively perceived by individuals through the recognition and evaluation of support from family members, friends, and important others (Fan et al., 2012). Researchers theorize that social support is one of the most useful stress buffers (Krysia and Wei, 2014). Some researchers have proposed that the reduction of social support produces a negative schema in depressed individuals, leading to cognitive bias in information processing and thus producing discrimination perceptions (Zhang et al., 2019). According to the buffering model of social support states, perceived social support is a protection mechanism that can buffer the negative impact of negative stimuli on an individual, allowing the individual to avoid all kinds of negative emotions (Aneshensel and Stone, 1982; Etzion, 1984; Tiegs, 2010). Empirical studies have also found that social support can reduce the perceived discrimination of an individual (Wang and Zhang, 2020). Therefore, if a certain method can be used to improve the perceived social support of an individual, the perception of discrimination may be significantly reduced. One study conducted a social support skill-training group intervention to treat veterans with post-traumatic stress disorder and found a positive effect of this training (Sirati-Nir et al., 2018). Another study adopted a 13-week group treatment intervention focusing on social skills training and cognitive restructuring and found that the intervention increased the perceived social support of participants from family (Brand et al., 1995). However, although these interventions are effective, most of them require long-term treatment. Thus, in the context of COVID-19, these interventions may be difficult to implement. Therefore, it is necessary to find more concise and effective interventions.

Wise intervention is a new intervention method developed in recent years. Unlike previous interventions, it aims to change the way people feel and think in their lives and has a low resource and time investment but long-term effects (Walton and Wilson, 2018). These interventions are very much like an everyday experience, and their purpose is simply to change the specific way people think or feel in their normal lives to help individuals thrive (Walton, 2014). The wise intervention has prominent advantages over other intervention methods. First, wise intervention holds that the individual psychological process does not work in a vacuum but in a complex system. Therefore, it is more suitable for the specific situation facing individuals and can promote the self-reinforcement of individual thoughts and behaviors over time (Walton and Cohen, 2011). Second, wise intervention is characterized by simplicity, accuracy, and strong operability (Logel and Cohen, 2012). Third, wise intervention does not have additional negative effects and requires less time and resources. This intervention method has increasingly been applied to different areas of social life, such as education (Yeager et al., 2013), close relationships (Finkel et al., 2013), and mental health (Peng, 2019), and has achieved remarkable results.

The number of people affected by the COVID-19 epidemic is so large that researchers need to consider exploring interventions that are easy to implement in specific situations such as quarantining at home. Therefore, based on the idea of wise intervention, this study chooses perceived social support as the intervention point by referring to the influencing factors of perceived discrimination, the view of the buffer model, and the relevant theoretical and empirical research results. That is, through perceived social support, wise intervention is used to help college students who returned home from Wuhan think about their plight from multiple perspectives, understand their environment, improve perceived social support, and thus reduce perceived discrimination.

Wuhan, the capital city of Hubei Province, China, the initial epicenter of COVID-19, was put under an international spotlight, leading to the stigmatized label “Wuhan virus” (Yang et al., 2020). Such COVID-19-related discrimination was quite evident and omnipresent among individuals, especially those who manifested a potential linkage with Wuhan during the outbreak of COVID-19 since Wuhan was the first epicenter of this global health crisis (Li et al., 2020). On January 23, 2020, the Chinese government locked down Wuhan in an unprecedented effort to curb the spread of COVID-19 (Xinhua Net, 2020), and Hubei Province 2 days later. And after February 21, there was no explosive growth of daily confirmed infections in any Chinese province except Hubei Province (Ye and Lyu, 2020). Thus, people from Wuhan or Hubei Province were targeted and blamed by other Chinese people (Ren et al., 2020). Therefore, this study will first use the questionnaire method to determine the perceived discrimination of college students returning home from Wuhan during the epidemic and then use the wise intervention program to intervene for the individuals who feel discriminated against because of the situation. We also investigate the potential mechanism of this intervention method.

## Study 1. Investigation of Perceived Discrimination

In China, the rapid spread of COVID-19 in the early stages of the outbreak was mainly due to the large number of people returning home to meet their families during the Spring Festival (Liu et al., 2020), including thousands of college students studying in Wuhan. Wuhan, as one of the key higher education hubs of China, hosts a large number of university students, amounting to 1.3 million (One in 10 Wuhan residents is a university student) (Yang et al., 2020). Coming from every province of China, the majority of these students returned to their hometown in mid-January during the winter vacation. Some of the students who studied in Wuhan returned to their hometowns in other cities within Hubei Province, which were also severely affected by the epidemic. Some returned to their hometowns outside Hubei Province, where the epidemic situation was relatively mild. Soon after the students returned home, intensive anti-epidemic measures were put in place across the country, and the students who returned from Wuhan became the focus of quarantine and anti-epidemic efforts. Because they were treated differently, it is possible that they perceived discrimination (Major et al., 2002). And it is also possible that the perceived discrimination was different by hometown address (within Hubei Province *vs*. outside Hubei Province).

### Methods

#### Participants

In March 2020, when the general public was ordered to quarantine at home, we enrolled college students returning home from Wuhan to complete the questionnaire. Data were collected using an online convenient questionnaire tool (https://www.wjx.cn/). In the context of COVID-19 quarantine, convenience and snowball sampling methods were used to recruit the participants, that is, five researchers (students in Hubei University) first distributed the questionnaire link through their social media communication group (QQ group), and then invited the participants to forward the questionnaire link to more college students returning from Wuhan. Only fully completed questionnaires could be successfully submitted online. It was made clear that the participation was voluntary. Participants could withdraw at any time for no reason by simply closing the questionnaire page. Ultimately, a total of 382 questionnaires were received, one invalid questionnaire with all items filled with the same answers was eliminated, and 381 valid questionnaires were obtained (questionnaire recovery efficiency, 99.7%). The participants were aged between 17 and 24 years old (mean age 20.17, SD = 1.55), including 120 male college students and 261 female college students. There were 168 college students returning home within Hubei Province and 213 students returning home outside Hubei Province. The study was approved by the Ethical Committee of the Institute of Education, Hubei University. Individuals who agreed to participate were given information about the study, and informed consent was obtained from the students or their parents, for individuals under the age of 18.

#### Measures

##### Perceived Personal Discrimination Scale

The questionnaire was developed by Shen et al. (2009), which is a 2-dimensional scale used to assess the perceived discrimination (Liu and Shen, 2010). The questionnaire included six items dividing into 2 dimensions (individual discrimination perception and group discrimination perception). Three items were used to measure individual discrimination perception, such as “I feel I have been treated differently”; and three other items measured group discrimination perception, such as “On the whole, people with similar background and experiences like me have been treated unfairly.” The responses of the participants in each item were recorded on a 5-point Likert scale ranging from 1 (strongly disagree) to 5 (strongly agree). The higher the total score, the more intense the perceived discrimination. This questionnaire is widely used in China and shows good validity and reliability (Shen et al., 2009; Liu and Shen, 2010; Zhang et al., 2019). We revised the questionnaire in this study to fit the context of the epidemic situation. The main change is to add background restrictions (i.e., as a person returning home from Wuhan after the outbreak of the epidemic). The Cronbach's alpha coefficient for this study is 0.88.

#### Data Analysis

For data analysis, we used the statistical package SPSS 22.0 for Windows. Data were described using the mean and SD for continuous data. A *p* ≤ 0.05 was considered significant. During the outbreak of COVID-19, there were great differences in the severity of the epidemic and the acceptance of Wuhan returnees between Hubei Province and other provinces. Therefore, we further used an independent sample *t*-test to explore the differences in perceived discrimination by hometown address (within Hubei Province *vs*. outside Hubei Province).

#### Results

**Figure 1:**
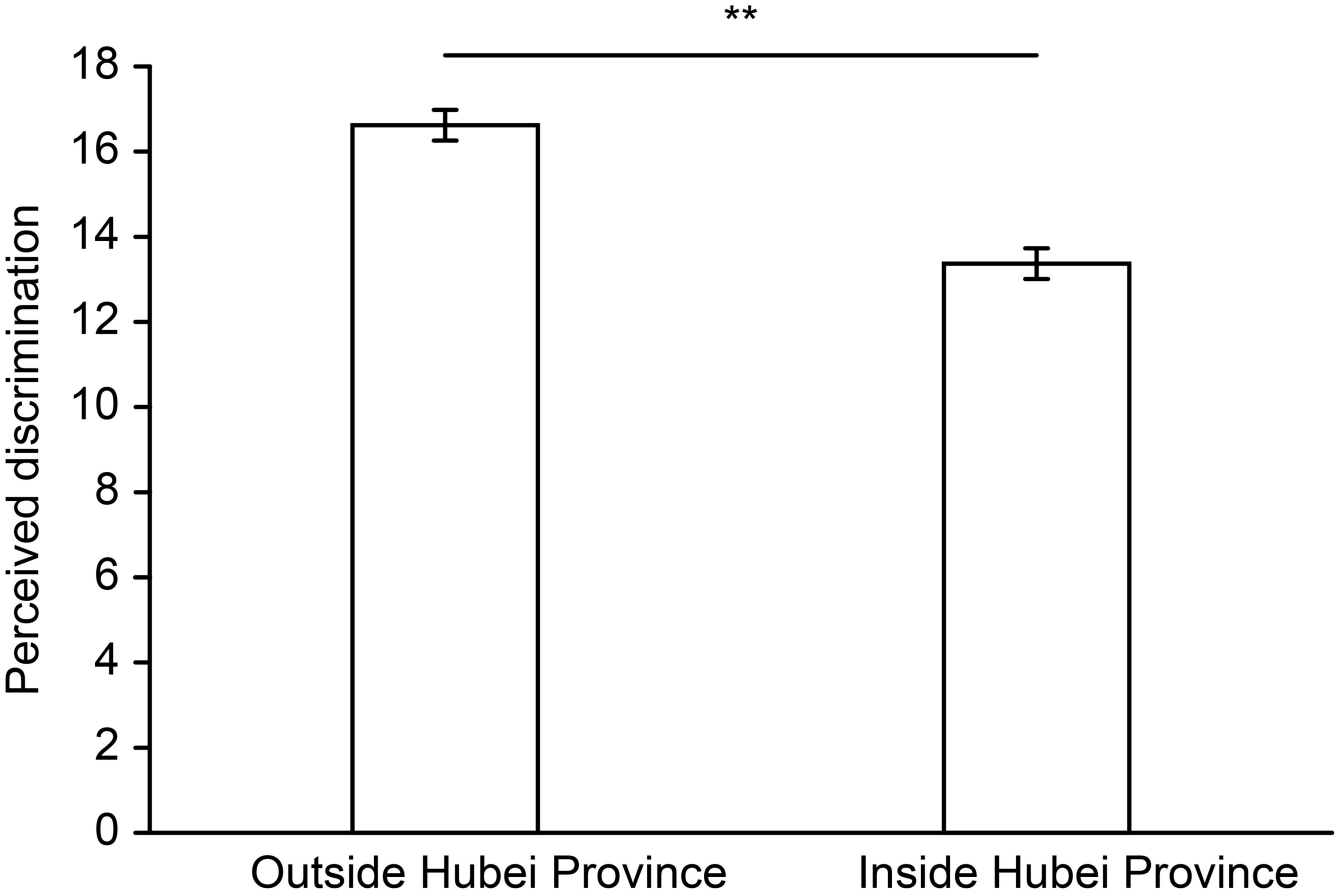
Perceived discrimination by hometown address (error bars show ± 1 SEM, **p < 0.01).

The results showed that the perceived discrimination score of college students returning home from Wuhan was 15.19 ± 5.24, with the highest score being 30 and the lowest score being 6. The comparison of perceived discrimination among students who returned home from Wuhan by hometown address is shown in Figure 1. For those with hometown addresses outside Hubei Province, the perceived discrimination score was 16.62 ± 5.23, which was significantly higher than the theoretical median value of 15, *t* (212) = 4.55, *p* < 0.01, *Cohen's d* = 0.62. For the students who returned to towns within Hubei Province, the perceived discrimination score was 13.37 ± 4.67, which was significantly lower than the theoretical median value of 15, *t* (167) = −4.51, *p* < 0.01, *Cohen's d* = −0.70. Moreover, there was a significant difference in perceived discrimination between the two groups, *t* (379) = 6.32, *p* < 0.01, *Cohen's d* = 0.65.

#### Discussion

The results showed that college students returning from Wuhan to towns outside Hubei Province from Wuhan perceived significantly more discrimination. Considering the actual situation of the epidemic outbreak at that time and the cause of perceived discrimination, a possible reason for this greater perception of discrimination reports in the media that most infected patients in other regions had some connection to Wuhan, either by traveling to Wuhan or by contacting infected patients in Wuhan (Publicity Department of the Central Committee of the Chinese Communist Party Home Page, 2020). The Chinese government quarantined the city of Wuhan on January 23, 2020, and Hubei Province 2 days later. Therefore, in Hubei Province, people felt that “misery loves company.” As a result, people who had been to Hubei were subject to more serious stigma and xenophobia (Moukaddam and Shah, 2020), resulting in greater perceived discrimination for college students who returned to towns outside Hubei Province. As the stress of perceived discrimination negatively affects mental and physical health (Williams et al., 1997), it is necessary to provide interventions to those who have perceived discrimination. Therefore, we will further intervene in the perceived discrimination of college students returning from Wuhan to towns outside Hubei Province.

## Study 2. Wise Intervention on Perceived Discrimination

The results of Study 1 showed that the level of perceived discrimination of college students returning from Wuhan to other provinces was relatively high. This study intervened with these people. To reduce perceived discrimination and prevent possible psychological problems, this study adopted the concept of wise intervention, created intervention materials for perceived social support, and intervened in the form of reading and writing.

The purpose of this study is to determine whether the intervention is effective and to identify its mechanism. Based on previous studies, we hypothesize that (1) wise intervention for perceived social support can significantly affect the perceived discrimination of college students returning home from Wuhan and that (2) perceived social support plays a mediating role in the influence of the intervention on perceived discrimination.

### Methods

#### Participants

From March to April 2020, college students returning from Wuhan to provinces outside Hubei Province were recruited through the Internet to participate in the intervention experiment. A total of 79 participants participated, and 16 participants who did not complete the experiment as required were excluded. The high dropout percentage might be because the experiment was launched online. In the context of the epidemic, we could not conduct the experiment in the laboratory. We also made it clear that the participation was voluntary and they can withdraw from the study at any time without providing a reason. The participants might feel less pressure to withdraw from the experiment online. Furthermore, the total experiment took ~40 min to complete, the time is relatively long, so some subjects quit before they have completed the experiment. Finally, 63 participants (15 males and 48 females) completed the experiment. There were 31 participants in the experimental group and 32 participants in the control group, all aged between 18 and 23, with an average age of 20.33. The age difference between the two groups was not significant, *t* (61) = −0.52, *p* = 0.607, and the sex difference was not significant, χ^2^= 0.13, *p* = 0.714.

G^*^Power version 3.1 (Faul et al., 2007) was used to compute the required sample size of the experiment. With effect size *f* set at 0.25, alpha set at 0.05, two groups, and two repetitions, correlation among repetitions 0.5, non-sphericity correction = 1 yield a total sample size of 54 for testing the within-between interaction hypothesis, with 27 subjects in each group. In the present study, the final sample size was 31 participants in the intervention group and 32 in the control group. A bigger sample size than required would lead to a greater statistical power.

#### Measures

##### Perceived Personal Discrimination Scale

This scale is the same as that used in Study 1.

##### Perceived Social Support Scale

The PSSS was developed by Zimet et al. (1988). We used the Chinese version, revised by domestic scholar Jiang (1999), to measure the degree of perceived social support. There are 12 items on the scale, which are divided into three dimensions: family support, friend support, and other support. All items are rated on a 7-point Likert scale (1 = strongly disagree, 7 = strongly agree). A lower score indicates poorer social support. The Chinese version of the PSSS has demonstrated good reliability in prior studies (Cronbach alpha = 0.88) (Huang et al., 1999). In this study, we changed “leader, relative, and colleague” to “community, neighbor, and volunteer.” Combined with the special social situation during the epidemic period, we change the original “leaders, relatives, and colleagues” to “community, neighbors, and volunteers.” The Cronbach's alpha coefficient for this study is 0.91.

#### Procedure

**Figure 2:**
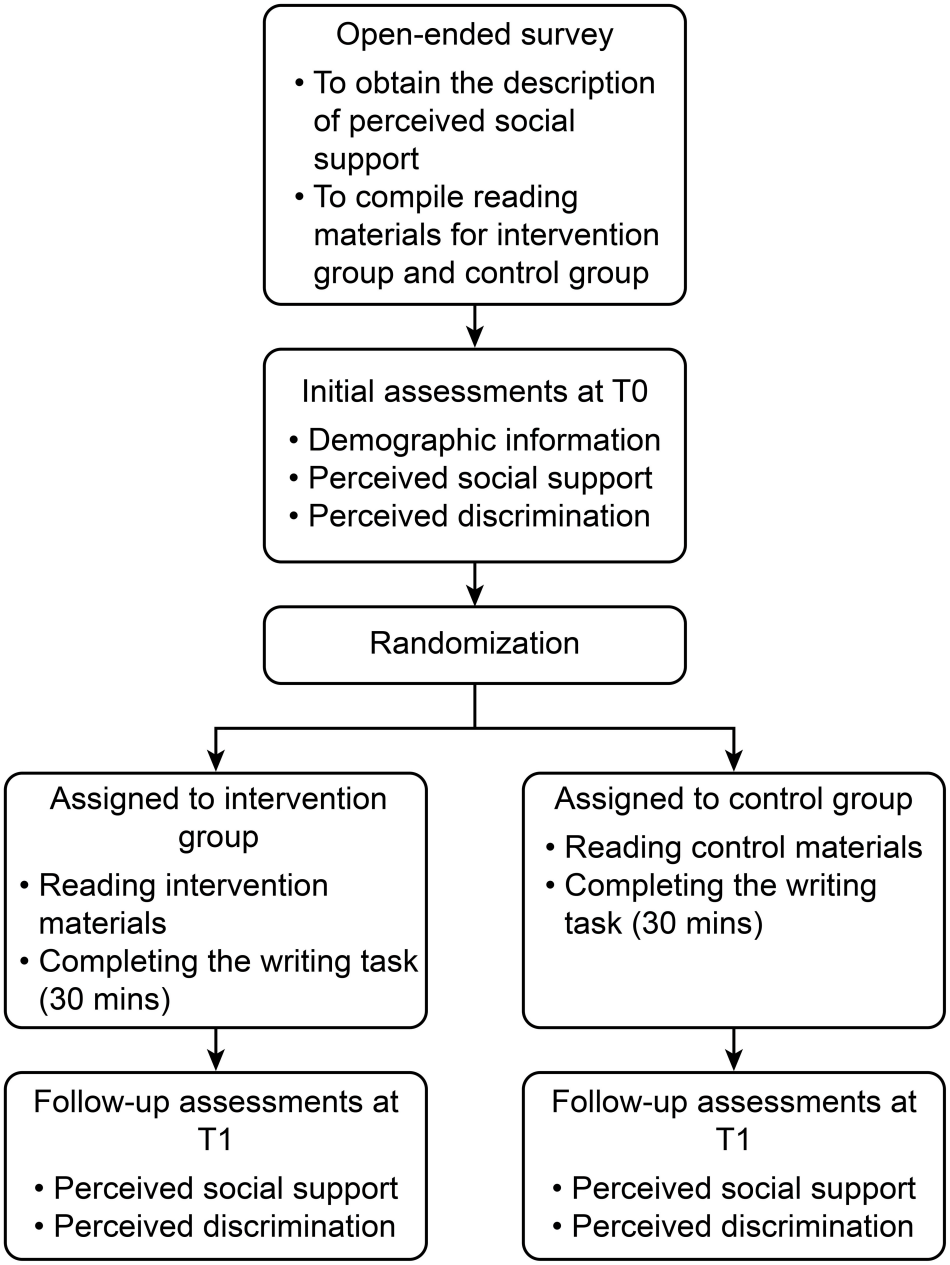
Diagram of the study cohort.

There are four stages in the present study: an open-ended survey, an initial assessment, intervention, and a follow-up test (Figure 2).

##### Open-Ended Survey

Before the intervention, we used an open-ended questionnaire to determine the actual situation of students returning home from Wuhan to prepare the reading materials for the experimental group. To prevent contamination, no participants who participated in the open-ended survey were allowed to participate in the formal experiment, and they were required not to inform others of the experimental contents.

First, five trained psychology undergraduates compiled the first draft of the perceived social support open-ended questionnaire by reading literature and having many rounds of discussion. Then, the questionnaire was sent to 23 psychology undergraduates who did not help compile the questionnaire, and they provided some modification suggestions. After amendments, the questionnaire was validated by three professional teachers of psychology. Finally, the questionnaire was sent to college students who did not major in psychology to ensure that the questionnaire was unambiguous.

The structure of the final open-ended questionnaire was as follows: (1) To understand the difficulties of the participants after returning home as a whole. For example, “What difficulties did you experience, and what did you worry about when you came back from Wuhan during the epidemic? How have these difficulties and concerns changed over time?” (2) They were asked to write about their experiences of perceived social support in terms of family support, friend support, and other support. For example, “How did you get on with your family after you came back from Wuhan during the epidemic? What did your family do to make you feel positive or negative about yourself or your hometown? Please provide enough details so that other students returning from Wuhan can understand your experience. Has your relationship with your family changed over time? If so, what has changed your relationship with your family?”

After the questionnaire was constructed, it was distributed to 18 college students returning home from Wuhan (9 male students and 9 female students, aged 20.33 ± 2.09 years). According to the answers of the participants, the researchers integrated them into a positive social support intervention material, which was used as the reading material for the experimental group in the intervention process. It should be noted that the positive meaning constructed in the intervention in this study is not a reversal of black and white because the researchers constructed the positive meaning against the ambiguous situation (rather than a situation with overt hostility), striving to help the individuals who returned home from Wuhan acquire adaptive meaning during a critical period.

The final reading materials of the experimental group included “the experience of other college students returning home from Wuhan” and “blessings and encouragement from other college students returning home from Wuhan.” In the part of “the experience of other college students returning home from Wuhan,” the positive social support examples were integrated into three parts: family support, friend support, and other support. For example:

“At first, I was worried that I was a potential carrier of the virus; when I came back, I was scared and worried that I was going to get infected because of my little cough and worried that I was going to infect my family, but my family was still very nice to me. They didn't think I was going to get sick and treated me just like normal. When my neighbors discussed with my family that I had come back from Wuhan, my family would explain, “She came back early, the incubation period has passed, and it doesn't matter.” It makes me feel warm. When I have a fever, even though I have a common cold, my parents bring food and water to my bed to make me feel warm.” (Other experiences of college students returning home from Wuhan).

“During the quarantine period, because we couldn't get out of the house, our materials were purchased by the community. In the beginning, they were not proficient, and sometimes they made some small mistakes, but they took our suggestions actively and constantly improved. I can see the community workers were very busy and ate instant noodles for 3 days, and some got sick. Some students may be unhappy about tedious inspection reports, but it is their job duty, so we should cooperate with the community and together to overcome the outbreak. China refuels! Come on Wuhan!” (Other experiences of college students returning from Wuhan).

“Calm down and don't over blame ourselves. We didn't make mistakes. Quarantine is just to better control the epidemic. We are right to be isolated and contribute to the safety of the country, society, and others.” (encouragement and blessings from other college students returning from Wuhan).

“In fact, I think there will be anxiety at the beginning, and I will settle down slowly and face it with a peaceful mind. There are truly many people who care about you, and we should be good at discovering that everyone has goodwill. Let us overcome the epidemic together. Come on Wuhan!” (encouragement and blessings from other college students returning from Wuhan).

The reading materials allowed the participants to look at the events in the social situation from multiple perspectives, to see the things they were facing more positively in the current social background, and to change the meaning construction process of the original events, that is, to change the process of how people understood themselves and the social situation.

##### Initial Assessment

The initial assessment was used to evaluate the social demographic information of the participants (including the returning place, gender, and age) and the core variables of the study (including perceived social support and perceived discrimination). The initial assessment was taken at T0, which was right before the intervention.

##### Intervention

A wise intervention starts with a specific and well-founded theory. The accuracy of the theory allows researchers to create a precise tool instantiating the theory in short training sessions and altering a particular mental process in real life. In this study, according to social cognition approaches, perceived support is primarily a cognitive phenomenon that represents a highly abstracted and impressionistic view of the social world (Wang and Zhang, 2020). Therefore, through reading and writing, this intervention helps students returning home from Wuhan understand their environments by thinking about their plights from multiple perspectives, improve their perceived social support, and thus reduce the discrimination they perceive.

The participants were randomly assigned to the experimental group and the control group. The intervention lasted for ~30 min. Both groups were unaware of the difference in experimental conditions and did not know the specific experimental hypothesis.

The experimental group received perceived social support reading material, while the control group received non-intervention material. After reading the material, the participants were asked to complete the corresponding writing task (~300 words).

After reading the intervention material, each participant was given an instruction: “Please write a short essay based on your own experience. Tell about the people and things around you and their support and encouragement. In addition, leave what you would like to say to other college students returning home from Wuhan. We will select a part of the content of the composition to show to the next group of students returning home from Wuhan. I'm sure they will appreciate your efforts and your heart to get us through this difficult time.” In this task instruction, the participants were “supported and encouraged” to recall the process through further positive intervention, and the intervention information was internalized to play a better role. At the same time, the instruction emphasized “we will select a part of the content of the composition to show to the next group of college students returning home from Wuhan. I'm sure they will appreciate your efforts and your heart to help them to get through this difficult time.” We encouraged the participants to “consider themselves as the benefactor rather than the beneficiary” (Peng, 2019), to give strong meaning to the participation of the participants in this experiment and ensure their effective participation.

There are two important meanings of “writing” in this study. First, writing helped participants express their feelings and thoughts during the epidemic and further clarify their thoughts and feelings. Second, expressing the combination of their own experience and those of others in writing helped the participants view their social situation more positively and objectively.

The only difference in intervention procedures between the control group and the experimental group was the reading material. The reading materials of the control group were simple popular science articles, and the task after reading was to finish a series of writing tasks. The length and reading time of the articles were similar to those of the experimental group. To avoid excessive cognitive resource consumption and fatigue, short answers were given at the beginning of obvious paragraphs or underlined. This operation effectively controlled the experimental independent variables.

After the completion of the writing task, the participants were asked to take photos of the writing content and then send the photos to the experimenter to ensure that the participants completed the writing task seriously.

##### Follow-Up Assessment

When the participants finished their intervention task (T1), the main variables of this study (including perceived social support and perceived discrimination) were measured again using the scale that was used in the initial assessment.

At the end of the experiment, we sent the reading materials of the intervention group to the participants in the control group. They could choose whether to read or not according to their own needs. In addition, we provided all participants with the contact information of a psychological service hotline that can provide psychological assistance services during the epidemic period.

#### Data Analysis

SPSS 22.0 was used for data processing. The effectiveness of the intervention was analyzed by using a 2 × 2 repeated-measures (ANOVA, Greenhouse–Geisser corrections with corrected degrees of freedom), with time (T0 *vs*. T1) as a within-subject factor, and group (Intervention group *vs*. Control group) as a between-subjects factor. Then, independent *t*-tests and paired *t*-tests were performed, and perceived discrimination and perceived social support were compared with respect to group and time. The effect size estimates $\eta_{p}^{2}$ or *Cohen's d* were reported.

### Results

#### Baseline Assessment

The independent *t*-test results show that there were no significant between-group differences on any baseline outcome measure at T0: perceived discrimination: *t* (61) = −0.12, *p* = 0.909; perceived social support: *t* (61) = 0.15, *p* = 0.879.

The lack of a significant difference in perceived social support and perceived discrimination between the experimental group and the control group before the intervention indicates that the experimental group and the control group had good homogeneity and met the requirements of randomization before the intervention.

#### Intervention Effects

**Figure 3:**
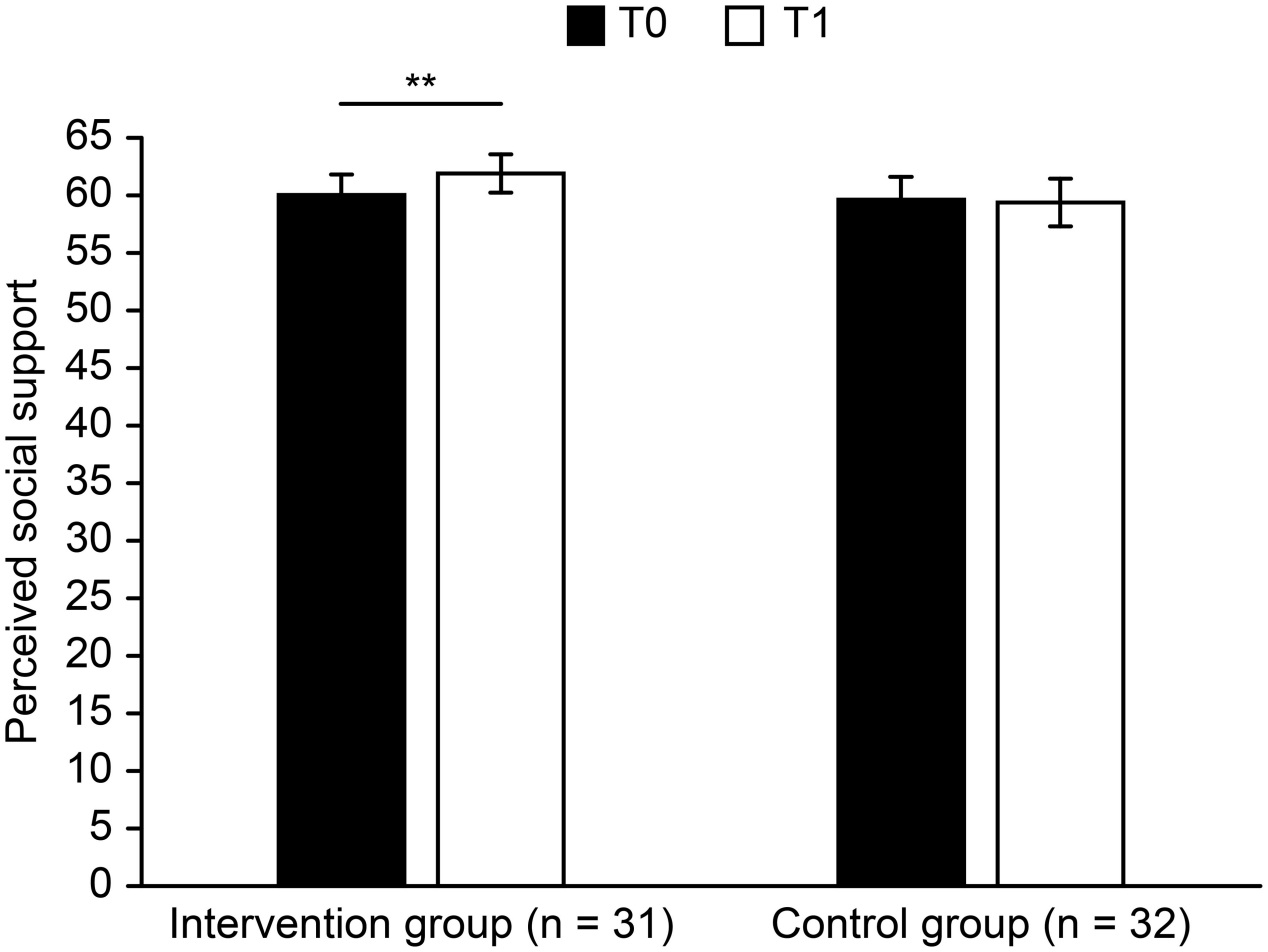
Perceived social support before and after intervention (error bars show ±1 SEM, **p < 0.01).

**Figure 4:**
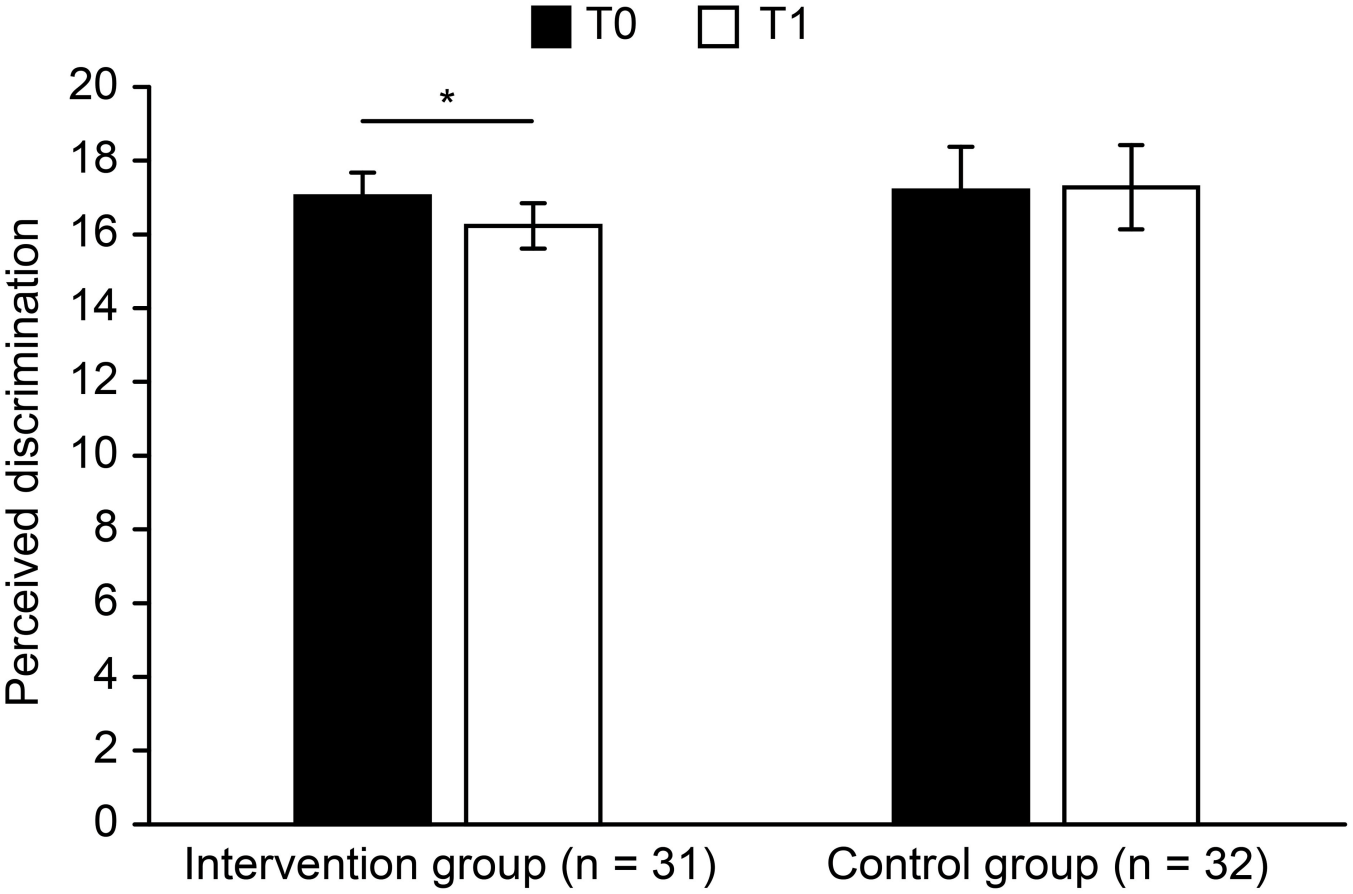
Perceived discrimination before and after intervention (error bars show ± 1 SEM, *p < 0.05).

To answer hypothesis 1, we performed a 2 × 2 ANOVA with time (T0 *vs*. T1) as a within-subjects factor and group (Intervention group *vs*. Control group) as a between-subjects factor. For perceived social support, the main effect of time [*F*_(1, 61)_ = 3.527, *p* = 0.065, $\eta_{p}^{2}$ = 0.055] was marginally significant, perceived social support (T0) > perceived social support (T1). The main effect of group [*F*_(1, 61)_ = 0.311, *p* = 0.579, $\eta_{p}^{2}$ = 0.005] was not significant. The interaction between time and group [*F*_(1, 61)_ = 6.039, *p* = 0.017, $\eta_{p}^{2}$ = 0.090] was significant. For the experimental group, perceived social support increased significantly (Figure 3), T0 *vs*. T1: *t* (30) = −2.92, *p* = 0.007, *Cohen's d* = −1.07. For the control group, there was no significant change in perceived social support, *t* (31) = 0.43, *p* = 0.670, *Cohen's d* = 0.15. For perceived discrimination, the main effect of time [*F*_(1, 61)_ = 1.865, *p* = 0.177, $\eta_{p}^{2}$ = 0.030] was not significant. No main effect of group [*F*_(1, 61)_ = 0.213, *p* = 0.646, $\eta_{p}^{2}$ = 0.003] was revealed. The interaction between time and group [*F*_(1, 61)_ = 2.976, *p* = 0.090, $\eta_{p}^{2}$ = 0.047] was marginally significant. For the intervention group, the perceived discrimination of the participants at T1 was lower than that of T0 (Figure 4), *t* (30) = 2.73, *p* = 0.010, *Cohen's* d = 0.99, while there were no differences for the control group, *t* (31) = −0.22, *p* = 0.828, *Cohen's* d = −0.08.

To investigate whether the intervention effectively decreased perceived discrimination, it is necessary to test whether there was a significant difference between the experimental group and the control group in the change in perceived social support or perceived discrimination after the implementation of the intervention (T1–T0). Independent sample *t*-tests were used to compare the changes in the experimental group and the control group, and the results showed that there was a significant difference in perceived social support between the two groups, *t* (61) = 2.46, *p* = 0.017, *Cohen's* d = 0.63, indicating that the intervention of the experimental group effectively increased perceivable social support level. The difference between the two groups was marginally significant on perceived discrimination, *t* (61) = −1.73, *p* = 0.090, *Cohen's* d = −0.44. This shows that compared with the control group, the experimental group experienced decreased perceivable discrimination.

#### The Mediating Effect of Perceived Social Support

To assess hypothesis 2, we tested the mediating role of perceived social support in the influence of the intervention on perceived discrimination. We used the Hayes (Hayes, 2013) PROCESS tool in SPSS. This widely used SPSS program is based on deviation correction of the percentile bootstrap method and can add intermediary regulation and adjustment to the variety of mediation model verifications.

The results are shown in Table 1. The intervention conditions had a marginally significant influence on perceived social support (*p* = 0.090, 95% *CI* = −3.36 ~ −0.05). After incorporating perceived social support as a mediating variable, the results showed that the intervention conditions had a significant influence on perceived social support (*p* = 0.017, 95% CI = −3.85 ~ −0.40). On perceived social support, the direct effect of intervention on perceived discrimination was marginally significant (*p* = 0.054, 95% CI = −0.30 ~ 0.00), while on perceived discrimination, the direct effect of intervention was not significant (*p* = 0.279, 95% CI = −0.48–1.65). It can be seen that perceived social support plays a complete mediating role in terms of the intervention conditions and perceived discrimination. The percentile bootstrap mediating effect test for bias correction further showed that (as shown in Table 2) the mediating effect value was 0.32, the 95% confidence interval of bootstrap was [−0.03, 0.95], and the mediating effect accounted for 35% of the total effect.

**Table 1:** Regression analysis of the mediating role of perceived social support.

**Dependent variable**	**Independent variable**	***R^**2**^***	***B***	***SE***	**β**	***t***	***p***
Perceived discrimination	Intervention condition	0.05	0.90	0.52	0.22	1.73	0.090
Perceived social support	Intervention condition	0.09	−2.12	0.86	−0.30	−2.46	0.017
Perceived discrimination	Intervention condition	0.10	0.59	0.53	0.14	1.09	0.279
	Perceived social support		−0.15	0.08	−0.25	−1.97	0.054

*Perceived discrimination and perceived social support were the changes before and after the intervention (T1–T0)*.

**Table 2:** The mediating effect of perceived social support.

	**Indirect effect value**	**Bootstrap standard error**	**Boot CI lower limit**	**Boot CI upper limit**	**Relative mediation effect**
Indirect effect	0.32	0.26	−0.03	0.95	35%

The results of Study 2 showed that the wise intervention program had a significant effect on the perceived discrimination of college students returning home from Wuhan, which proved that the intervention method used in this experiment was effective. We also found that while the perception of discrimination decreased, the perceived social support of individuals increased. Therefore, Study 2 further verified the mediating effect of perceived social support between intervention conditions and perception of discrimination, and the results showed that perceived social support played a complete mediating effect. Hypothesis 2 was confirmed.

## General Discussion

In the context of the COVID-19 outbreak, the mental health of the individuals has attracted much attention. Some studies have investigated the mental health of college students in China and found that the mental health level of Chinese college students was affected by the epidemic (Chen et al., 2020; Wang et al., 2020). Based on this, we took college students returning home from Wuhan as the subjects and found that these college students returning home from Wuhan to other provinces experienced more perceived discrimination. Then, we proposed a wise intervention method to intervene in the perceived discrimination perception of these college students and found that the wise intervention effectively reduced the perceived discrimination of college students returning home from Wuhan.

In the investigation of the mental health status of college students returning home from Wuhan, we found that their perceived discrimination was higher than the norm and was significantly affected by hometown location (within Hubei Province *vs*. outside Hubei Province): the perceived discrimination of students returning to towns outside Hubei Province was significantly higher. Considering the detailed experience shared by the participants in Study 2, there are several possible reasons: (1) The epidemic was more severe in Hubei Province than in other provinces. COVID-19 is reported to spread mainly through respiratory droplets, direct contact, aerosol diffusion, and so forth, and these modes of transmission are closely related to population mobility (Center for Disease Control and Prevention Home Page, 2020; Jiang and Luo, 2020). On January 27, 2020, all cities within Hubei Province reported confirmed COVID-19 cases (Jiang and Luo, 2020). Since then, all the people from Hubei Province have become the targets of protection. Therefore, college students returning from Wuhan to their hometowns within Hubei Province may not have experienced being treated differently. However, in areas outside Hubei Province, where the epidemic was less severe, stricter measures were taken to monitor those who returned from Wuhan. Under these circumstances, college students who returned from Wuhan to other provinces became “disadvantaged groups.” According to the theory of relative deprivation in social comparison theory (Mummendey et al., 1999), members of disadvantaged groups often experience the feeling of being deprived of their basic rights. This sense of deprivation would make college students returning from Wuhan more likely to perceive discrimination. (2) The perceived social support of college students returning from Wuhan to other provinces was low, which ultimately led to higher perceived discrimination. When college students returned from Wuhan to their hometowns outside Hubei Province, instead of being welcomed by relatives and friends, they were met with defensiveness and rejection, and their perceived social support was severely reduced, leading to more perceived discrimination. Studies have shown that poor received and perceived social support negatively influences the mental health of people (Vaingankar et al., 2020). By contrast, perceived social support is generally beneficial to an adaptation of the individual. The more social support an individual receives, the better his/her adaptation will be (Chirkov et al., 2008). In conclusion, this suggests that when the COVID-19 outbreak occurred, people may have adopted a series of treatment with negative bias toward those who had been to the epidemic area, leading to strong perceived discrimination among those who returned from the epidemic area and possibly mental illness (Evans-Lacko et al., 2012). Therefore, adopting some methods to increase perceived social support of people may help them avoid some adverse consequences.

This study also found that perceived social support played a mediating role between intervention conditions and perceived discrimination; that is, by enhancing individual perceived social support, the perception of discrimination among college students returning from Wuhan was reduced. According to the stress-buffering model, social support protects mental health by buffering the effect of perceived discrimination (Cohen and Wills, 1985; Krysia and Wei, 2014).

In line with the primary hypothesis, wise intervention for perceived social support can significantly affect the perceived discrimination, which manifested as a large effect (*Cohen's d* = −1.07). This study asked the participants to read the detailed experience of other college students who returned home from Wuhan and to complete the corresponding writing tasks. Showing examples of the student of support from relatives, friends, and society from different angles changed their understanding of their current social situations, enhanced their understanding of their social support level, and changed their idea that “other people exclude me because of the bad situation and are unwilling to help me,” which reduced the discrimination they perceived. This is consistent with previous research results; that is, social support can reduce the perception of discrimination of an individual (Podsakoff et al., 2012; Zhang et al., 2019), and perceived emotional support from family in response to a serious problem buffer the stress caused by high levels of everyday discrimination (Krysia and Wei, 2014). In addition, other researchers have proposed that the more social support an individual receives, the less lonely he or she feels, and the more positive emotions he or she experiences (He et al., 2015; Wood and Cook, 2019). Therefore, perceived social support may be an important protective resource in the context of epidemic situations and may effectively reduce the perceived pressure on individuals, thus protecting their psychological well-being.

In addition, we used a new concept of intervention and proposed a wise intervention for perceived social support. We used reading and writing to intervene in the perceived discrimination of an individual. Previous studies have confirmed that epidemic outbreaks have been historically accompanied by stigma, discrimination, and xenophobia, leading to psychological harm to individuals in the epicenter (Villa et al., 2020). Therefore, psychological interventions that reduce the perceived discrimination in the people exposed to the pandemic could be helpful in preventing the development of mental illnesses. In this study, the wise intervention method effectively reduced the perceived discrimination of college students returning home from Wuhan. This method is different from traditional long-term interventions, with the advantages of being short-term, low-cost, and more concise. Moreover, this method has lower requirements for the implementers of the intervention and can be completed over the Internet (Walton and Cohen, 2011; Logel and Cohen, 2012). Currently, the epidemic has spread to a pandemic, emphasizing the importance of managing psychological problems. We can consider promoting the use of this method to reduce perceived discrimination among infected people, suspected infected people, and other people who experience discrimination by others to reduce the possibility of psychological problems.

Finally, some limitations of the study deserve noting. (1) In the context of COVID-19 quarantine, convenience, and snowball sampling methods were used to recruit the sample. As the study did not limit the gender of the subjects, the final results showed that the majority of the sample in this study were female subjects (accounted for 68.5% of the total sample). This limits the generality and generalization of the conclusions, so future studies should balance gender ratios and verify the results of this study in a wider population. (2) The original plan of this study was to use multiple time points for the posttest to evaluate the effectiveness of this wise intervention method. However, this experiment is closely related to the epidemic environment. The Chinese government took strong anti-epidemic measures so that the epidemic situation could be better controlled. Therefore, the psychological states of the people changed greatly with changes in the social situation, so this study failed to conduct multiple posttests. However, the core idea of the intervention is to “change the meaning construction of the participants,” which requires the participants to continuously internalize and thus produce long-term effects. Therefore, in the future, researchers can design multiple posttests in a standardized environment to test the long-term effects of this intervention.

## Conclusions

In this study, we found that during the COVID-19 outbreak, college students returning from Wuhan to their hometowns outside Hubei Province (areas where the epidemic was not severe) perceived discrimination. Therefore, we used the wise intervention program for perceived social support to assist these college students. The results showed that this intervention program effectively reduced the perceived discrimination of college students. In addition, this study further explored the potential mechanism of the effect of the intervention. It was found that the intervention reduced the perceived discrimination of individuals by improving their levels of perceived social support.

The results of this study provide some insight into the prevention and intervention for mental health problems among individuals affected by the epidemic. Specifically, the content of intervention (perceived social support) and the form of intervention (wise intervention) can be designed to prevent the occurrence of psychological problems in epidemic situations. Moreover, the investigation of the mechanism of intervention is helpful for preventing or intervening in the discrimination perceived by individuals and helps develop more effective intervention programs.

## Data Availability Statement

The raw data supporting the conclusions of this article will be made available by the authors, without undue reservation.

## Ethics Statement

The studies involving human participants were reviewed and approved by the Ethics Committee of Hubei University. Written informed consent to participate in this study was provided by the participants or the participants' legal guardian/next of kin.

## Author Contributions

TL, ZR, and WY: conceptualization. TL and LW: data curation and resources. ZG and XZ: formal analysis. HL, XZ, and LH: investigation. TL and ZR: methodology and validation. WY: project administration. ZR: supervision. ZG, HL, and XZ: writing—original draft. TL, ZG, ZR, and WY: writing—review and editing. All authors contributed to the article and approved the submitted version.

## Conflict of Interest

The authors declare that the research was conducted in the absence of any commercial or financial relationships that could be construed as a potential conflict of interest.
