# Supplementary material for: Effects of Wise Intervention on Perceived Discrimination Among College Students Returning Home From Wuhan During the COVID-19 Outbreak
Source: Front Psychol. 2021 Jun 7;12:689251. doi: 10.3389/fpsyg.2021.689251 (PMC8215144; doi:10.3389/fpsyg.2021.689251)
Supplement: Supplementary file 1 [file Table_1.DOCX]

**Perceived Personal Discrimination Scale (1= strongly disagree, 5 = strongly agree)**

| 1 | Since the outbreak of the epidemic, as a person who has returned from Wuhan, I feel that I have been treated differently. | 1 | 2 | 3 | 4 | 5 |
| --- | --- | --- | --- | --- | --- | --- |
| 2 | Since the outbreak of the epidemic, as a person who returned home from Wuhan, on the whole, people with similar background and experiences like me have been treated unfairly. |  |  |  |  |  |
| 3 | Since the outbreak of the epidemic, as a person from Wuhan back home, I feel that I am looked down upon. |  |  |  |  |  |
| 4 | Since the outbreak of the epidemic, as a person who returned home from Wuhan, on the whole, people with similar experiences and backgrounds with me have been looked down upon. |  |  |  |  |  |
| 5 | Since the outbreak of the epidemic, as a person who has returned home from Wuhan, on the whole, people with similar experiences and backgrounds to mine have been treated differently. |  |  |  |  |  |
| 6 | Since the outbreak of the epidemic, as a person who returned home from Wuhan, I feel that I have been unfairly treated. |  |  |  |  |  |

**Perceived Social Support Scale (1 = strongly disagree, 7 = strongly agree)**

| 1 | When I come across something, some people (community, neighbors, volunteers) will show up around me | 1 | 2 | 3 | 4 | 5 | 6 | 7 |
| --- | --- | --- | --- | --- | --- | --- | --- | --- |
| 2 | I can share happiness and sadness with some people (community, neighbors, volunteers) |  |  |  |  |  |  |  |
| 3 | My family can really help me. |  |  |  |  |  |  |  |
| 4 | I can get emotional help and support from my family when I need it. |  |  |  |  |  |  |  |
| 5 | When I am in trouble, some people (community, neighbors, volunteers) are the real source of comfort. |  |  |  |  |  |  |  |
| 6 | My friends can really help me. |  |  |  |  |  |  |  |
| 7 | I can rely on my friends in times of difficulty. |  |  |  |  |  |  |  |
| 8 | I can talk to my family about my problems. |  |  |  |  |  |  |  |
| 9 | My friends can share happiness and sadness with me. |  |  |  |  |  |  |  |
| 10 | In my life, some people (community, neighbors, volunteers) care about my feelings. |  |  |  |  |  |  |  |
| 11 | My family is willing to assist me in making decisions. |  |  |  |  |  |  |  |
| 12 | I can discuss my problems with my friends. |  |  |  |  |  |  |  |
